# Relationship Between COVID-19 and Linezolid-Resistant Enterococci: A Retrospective Single-Center Study

**DOI:** 10.7759/cureus.57227

**Published:** 2024-03-29

**Authors:** Amber Kumar, Akhil Taneja, Yogendra Pal Singh, Gaurav Pratap Singh, Saurabh Jain, Suchitra Jain

**Affiliations:** 1 Critical Care Medicine, Max Super Specialty Hospital, Delhi, IND; 2 Microbiology, Max Super Specialty Hospital, Delhi, IND

**Keywords:** enterococcus faecium, enterococcus faecalis, vancomycin resistant enterococci, multidrug resistance, antimicrobial resistance

## Abstract

Aim and objectives: To evaluate the correlation between whether the COVID-19 pandemic turned out to be a great premise for increasing the incidence of linezolid resistance infections.

Materials and method: The current retrospective study included data from March 2018 to March 2023 from a single center. The clinical records of the patients were reviewed to extract clinical data. Data gathered from medical records included demographic information, the type of specimen taken, the organism identified, and its sensitivity. Antibiotic susceptibility testing and bacterial identification are both done using the fully automated VITEK system.

Results: The total number of samples collected in all the groups, i.e., Group 1 (PRE-COVID), Group 2 (COVID), and Group 3 (POST-COVID), were 201, 127, and 1315, respectively. Out of a total of 201 samples in Group 1, i.e., from March 2018 to February 2020, 47 (23.38%) samples were collected from blood, 104 (51.74%) samples were collected from urine, and the rest of the samples were collected from other sources (pus, sputum, wound, stool, pleural fluid, etc.). In Group 2, i.e., from March 2020 to February 2021, the total number of samples collected was 127, of which 21 were collected from blood, 86 were from urine, and the remaining 20 samples were from other sources. A total of 1315 samples were collected between March 2021 and February 2023, i.e., in Group 3, 598 samples were collected from blood and 548 samples from urine. The most common isolates in the study were *Enterococcus faecalis* (35.7%) and *Enterococcus faecium* (61.0%).

Conclusion: A new threat seems to be emerging in the era of COVID-19, the *Enterococcus* genus. Though the mechanism remains unidentified, the viral infection seems to cause changes in the bacterial flora, favoring *Enterococcus* and increasing gut permeability, which provides the perfect environment for *Enterococcus* bacteria to develop invasive infections. In our study, the prevalence of linezolid resistance was 18.2% for five years.

## Introduction

*Enterococci* are types of bacteria that are non-spore-forming, gram-positive *cocci*, and they generally emerge in short chains, *diplococci* (pairs), or as single ovoid (egg-shaped) cells. They are identified as catalase-negative, urease-positive, and Lancefield group-D antigen-positive [1].

They are common dwellers in the intestines of humans but can cause significant infections [2,3]. *Enterococcus faecalis *(Efs) and *Enterococcus faecium *(Efm) can cause severe infections, including urinary tract and soft tissues, and are involved in causing dangerous infections such as septicemia and meningitis [4]. They are also responsible for nosocomial infections or hospital-related infections such as sepsis, endocarditis, abdominal infections, etc. [5].

*E. faecium* belongs to a high-priority category that contains increasingly drug-resistant bacteria as per the WHO priority pathogen list, which includes the following pathogens: *E. faecium* (vancomycin-resistant), *Staph. aureus* (methicillin-resistant, vancomycin-intermediate, and resistant), *Helicobacter pylori* (clarithromycin-resistant), *Campylobacter* (fluoroquinolone-resistant), *Salmonella spp.* (resistant to fluoroquinolones), and *Neisseria gonorrhea* (resistant to third-generation cephalosporin and fluoroquinolones). Because of its capability to cause nosocomial infections and prevailing resistance to different antibiotics, *Enterococcus* species, especially *E. faecalis* and *E. faecium*, have emerged as notable clinical concerns. Understanding the nuances of *E. faecalis* and *E. faecium* behavior is crucial for devising effective strategies in the present landscape.

Linezolid is preferred as the primary choice antibiotic for infections like hospital-acquired pneumonia, complicated skin and skin structure infections (SSSIs), uncomplicated SSSIs, sepsis, and community-acquired pneumonia caused by *Staphylococcus* and *Enterococcus* groups [5]. It is the first man-made antimicrobial agent of the oxazolidinone class to be licensed for clinical use. It exerts its antibacterial action by inhibiting the formation of the 70s initiation complex and impacting its binding affinity with formylmethionyl-tRNA [5].

In 2000, Linezolid was approved by the American Food and Drug Administration (FDA) for vancomycin-resistant *enterococci* (VRE) infections [3,4]. Recently, linezolid was also recommended for the treatment of multi-drug-resistant tuberculosis infections [6].

Outbreaks of drug-resistant organisms often start in intensive treatment areas, where patients are at risk of developing infections with medication-resistant organisms. Most of these patients are treated empirically with broad-spectrum antibiotics [6]. Recently, with the prevalent use of linezolid, the escalating reports of linezolid-resistant gram-positive pathogens highlight the enhanced risk of linezolid resistance transmission [7].

Therapeutic resistance to linezolid is associated with a G2576T mutation in domain V of 23s ribosomal ribonucleic acid (rRNA) genes in *Enterococcus*, and the level of linezolid resistance is directly related to the number of 23s rRNA genes containing this mutation [5,8]. The most common factor among patients who develop linezolid-resistant *E. faecium* is therapy duration with linezolid [9].

The suggested modalities for the attainment of linezolid-resistant VRE are self-determining events of de novo selection of resistant mutants in colonizing or infecting VRE, possible patient-to-patient spread, and emergence of linezolid-resistant (LR) mutants from linezolid-intermediate vancomycin-resistant *Enterococci* during linezolid therapy [10].

The COVID-19 pandemic saw a rise in antibiotic usage due to the absence of specific antiviral therapies and clear directives. As viral infections like COVID-19 do not respond to antibiotics, their use has increased to manage secondary bacterial infections and the uncertainties surrounding the novel virus. Furthermore, fungal and bacterial co-infections in SARS-CoV-2 patients became novel difficulties for healthcare systems all over the world [11,12]. In many instances during the pandemic, antibiotic therapies were prescribed inappropriately, often without evidence of co-infection. This misuse contributed to concerns about antibiotic resistance and highlighted the importance of judicious prescribing practices, especially when facing viral infections like COVID-19 that do not necessarily require antibiotics.

Therefore, this research was undertaken to identify the change in antimicrobial resistance patterns and prevalence of linezolid-resistant *Enterococcus* before and after COVID-19 in our hospital.

## Materials and methods

The current retrospective study included data from March 2018 to March 2023 from a single center. The clinical records of the patients were reviewed to extract clinical data. This project was given the go-ahead by the community's research, ethics, and biosafety committee with an ethical number of IEC/2022/MAXP/235.

Data gathered from medical records included demographic information, the type of specimen taken, the organism identified, and its sensitivity. The findings of the antimicrobial susceptibility tests were interpreted using the guidelines of the Clinical and Laboratory Standards Institute (CLSI). Linezolid resistance was defined as MIC ≥ 8 mg/mL.

The samples that had a successful growth of *Enterococcus* were the ones that were used. Mistakenly labeled swabs, incorrect IDs that corresponded to the requesting form, inappropriate specimen containers, and swabs obtained from body areas that were not being investigated were some of the factors that led to the exclusion of samples.

Antibiotic susceptibility testing and bacterial identification are both done using the fully automated VITEK system. The records were divided into three groups: pre-COVID, COVID, and post-COVID. Group-1 (PRE-COVID) included cases between March 2018 and February 2020; Group-2 (COVID) included cases from March 2020 to February 2021; and Group-3 (POST-COVID) included cases between March 2021 and February 2023.

Statistical analysis

The information gathered was incorporated into an Excel sheet and further analyzed using IBM Corp. Released 2013. IBM SPSS Statistics for Windows, Version 22.0. Armonk, NY: IBM Corp. Quantitative (numerical variables) data was given as mean and standard deviation, whereas qualitative (categorical variables) data was provided as frequency and percentage. The ANOVA test was used to compare the three groups' mean values, while the chi-square test analyzed their frequency differences. If p≤0.05, it was considered statistically significant.

## Results

The total number of samples collected in all the groups, i.e., Group 1 (PRE-COVID), Group 2 (COVID), and Group 3 (POST-COVID), was 201, 127, and 1315, respectively. Out of a total of 201 samples in Group 1, i.e., from March 2018 to February 2020, 47 (23.38%) samples were collected from blood, 104 (51.74%) were collected from urine, and the rest of the samples were collected from other sources (pus, sputum, wound, stool, pleural fluid, etc.).

In Group 2, i.e., from March 2020 to February 2021, the total samples collected were 127, of which 21 were collected from blood, 86 were from urine, and the rest (20 samples) were from other sources. A total of 1315 samples were collected between March 2021 and February 2023, i.e., in Group 3, 598 samples were collected from blood and 548 samples from urine.

Various microorganisms found in the samples were *E. faecium*, *E. faecalis*, *E. avium*, and others, and their distribution is shown in Table 1.

**Table 1:** Comparison showing microorganisms found in samples collected in three groups ANOVA: Analysis of variance, *p≤0.05: Significant

Microorganism	Group 1	Group 2	Group 3	F-value	p-value
E. Faecium	129	77	701	415.54	0.001*
E. Faecalis	67	45	561	357.32	0.001*
E. Avium	3	3	6	234.12	0.001*
Others	2	2	47	478.98	0.001^*^

Table 2 shows the linezolid sensitivity of the samples collected. It includes samples that are susceptible, intermediate, or resistant to linezolid. Resistance to linezolid has increased after COVID-19.

**Table 2:** Descriptive statistics and comparison showing linezolid sensitivity on the samples collected ANOVA: Analysis of variance, p≤0.05: Significant

Linezolid	Group 1	Group 2	Group 3	F-value	p-value
Susceptibility	157	100	750	384.84	0.002*
Intermediate	6	2	128	246.43	0.001*
Resistance	38	25	438	583.56	0.001^*^

**Figure 1:**
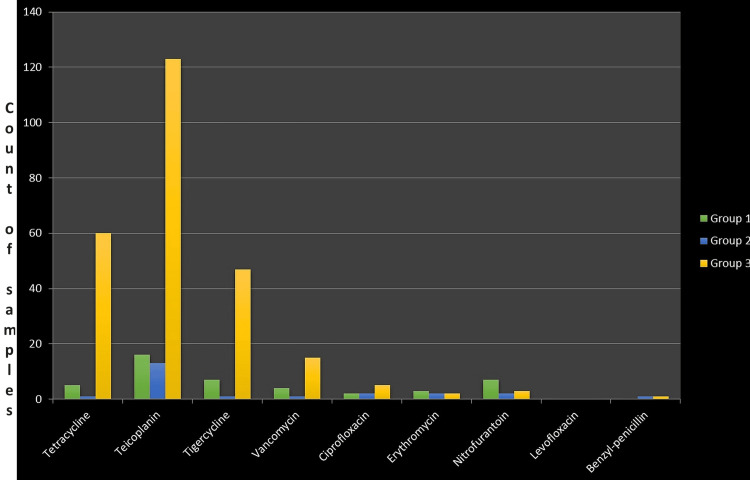
Drug susceptibility to linezolid resistance samples

Among all the drugs examined, the drug most sensitive to linezolid resistance samples is teicoplanin, followed by tigecycline, as shown in Figure 1.

## Discussion

The COVID-19 pandemic has begun the best assumptions for the progress of highly resistant bacterial strains due to the loss of antimicrobial use and the denial of proper, universally accepted protocols. The *Enterococcus* genus stands out as a uniquely identified bacteria in human infections. No surprise came when, during the pandemic, a high number of these types of infections were expected. However, the reason behind the heightened levels of *Enterococcus* in COVID-19 patients is still not fully understood. The intricate association between *Enterococcus* and SARS-CoV-2 needs further investigation, and researchers need to delve into the complexities and uncover the dynamics to provide valuable insights into the mechanism of infection and potential avenues for further therapeutic interventions.

Linezolid was introduced as a medicinal agent in the year 2000, with the primary focus on treating infections caused by resistant gram-positive *cocci*. Its approval marked a significant advancement in addressing antibiotic-resistant bacterial infections, particularly those caused by strains resistant to other antibiotics.

Regrettably, the extensive use of this antibiotic over the past 20 years has led to the occurrence of linezolid-resistant VRE in 2001 as well as an increase in the prevalence of these strains, particularly in hospitals [13,14].

Linezolid was recommended for VRE intravascular catheter-related bacteremia by the Infectious Diseases Society of America (IDSA); however, owing to considerable adverse effects such as myelotoxicity, its usage has been restricted, particularly in immunocompromised patients [15].

In *Enterococci*, one of the methods that grant confrontation to linezolid is the cfr gene, which encodes a multidrug-resistant protein [16]. This gene was initially identified in a strain of *Staphylococcus sciuri* that was derived from an animal. In *Enterococci*, it was initially documented from a strain of *E. faecalis* of animal origin.

The LEADER monitoring program in the United States found that the sustained susceptibility rate for linezolid was greater than 99.78% [17].

Linezolid, as an antibiotic, works well against gram-positive bacteria. As was mentioned before, it is unfortunate that laboratories in Europe have observed an increase in the occurrence of linezolid-resistant organisms [16]. This is a direct outcome of the increasing prevalence of antibiotic resistance [18] and perhaps not unexpectedly an association with relatively high numbers of patients admitted to intensive care units and death rates connected with such units [19]. There have been many reports of linezolid-resistant outbreaks in Ireland, with the intensive care unit (ICU) wards being the source of a major fraction of these infections [20,21].

Studies indicate that a significant proportion of *Enterococcus *isolates from human infections are attributed to *E. faecalis* [22]. In the respective study, the most common isolates were *Enterococcus faecalis *(35.7%) and *Enterococcus faecium* (61.0%), and the prevalence of linezolid resistance was 18.2% over five years.

Mališová et al. [23] found that *Enterococci* sent to the National Reference Laboratory for Antibiotics from 2009 to 2019 were resistant to linezolid. Other research work also found that the pattern of occurrence of linezolid-resistant *Enterococci* increased from year to year (0/2009-36/2019) [23,24].

Linezolid is one of the medications that should be used as a final option to treat infections of the skin and lower respiratory tract. It does this by inhibiting the process of protein synthesis in bacteria, which, in turn, prevents the development of bacteria [25]. There have been several cases of nosocomial outbreaks and infections triggered by *E. faecium* and *E. faecalis* strains that are sensitive to vancomycin as well as resistant to linezolid [16].

When compared to *E. faecium* strains, *E. faecalis* strains exhibit a linezolid resistance rate of 2.2%, which is much higher than the reported rate of 1.1% for *E. faecium* strains. The greatest rates of resistance from Linezolid-resistant *Enterococcus faecalis* and Linezolid-resistant *Enterococcus faecium *isolates were found in Africa and the United States of America, with 13.9 and 3.4%, respectively.

When compared to those obtained from water, *Enterococci* that were secluded from clinical samples had a significantly greater prevalence of antibiotic resistance. This conclusion should not come as a surprise because exposure to antibiotics occurs more often in hospital settings than in community settings.

The study's limitations include varying sample sizes across groups and potential biases in data collection. The study observed a notable increase in *Enterococcus* infections during the pandemic period. The prevalence of linezolid resistance, albeit at 18.2% over five years, underscores a concerning trend. However, the study's conclusion regarding the emergence of *Enterococcus* as a significant threat during COVID-19 remains speculative and warrants further investigation through prospective studies and molecular analysis to elucidate underlying mechanisms and establish causal relationships. Addressing these limitations and conducting more extensive research can provide a clearer understanding of antimicrobial resistance dynamics in the face of public health crises.

## Conclusions

In the era of COVID-19, a new concern has emerged: the *Enterococcus* genus poses a potential threat to the world. Now the focus is on a possible pathogen-to-pathogen link between SARS-CoV-2 and *Enterococcus*, which is affected by changes in the microbiome after bloodstream infections (BSI).

While the precise mechanism is still unclear, viral infections, such as SARS-CoV-2, appear to induce alterations in the bacterial flora. This shift favors *Enterococcus* and enhances gut permeability, creating optimal conditions for the development of invasive *Enterococcus* infections. Understanding these dynamics is crucial for devising strategies to manage associated health risks and improving our ability to manage and prevent associated infections.

## References

[1] García-Solache M, Rice LB (2019). The Enterococcus: a model of adaptability to its environment. Clin Microbiol Rev.

[2] Dadashi M, Sharifian P, Bostanshirin N (2021). he global prevalence of daptomycin, tigecycline, and linezolid-resistant Enterococcus faecalis and Enterococcus faecium strains from human clinical samples: a systematic review and meta-analysis. Front Med (Lausanne).

[3] Ruiz-Ripa L, Feßler AT, Hanke D (2020). Mechanisms of linezolid resistance among enterococci of clinical origin in Spain—detection of optrA- and cf.(D)-carrying E. faecalis. Microorganisms.

[4] Bi R, Qin T, Fan W, Ma P, Gu B (2018). The emerging problem of linezolid-resistant enterococci. J Glob Antimicrob Resist.

[5] Ma X, Zhang F, Bai B (2021). Linezolid resistance in Enterococcus faecalis associated with urinary tract infections of patients in a tertiary hospitals in China: resistance mechanisms, virulence, and risk factors. Front Public Health.

[6] Samantaray S, Deepashree Deepashree, Cherian A, Anitha Anitha, Sastry A (2020). Linezolid resistant Enterococcus faecium in sepsis. Indian J Microbiol Res.

[7] Dembicka KM, Powell J, O'Connell NH, Hennessy N, Brennan G, Dunne CP (2022). Prevalence of linezolid-resistant organisms among patients admitted to a tertiary hospital for critical care or dialysis. Ir J Med Sci.

[8] Sami H, Singh A, Ahmed S, Shahid M (2020). Emergence of linezolid resistance in Enterococci- prevalent genotype and resistance pattern in vancomycin-resistant enterococci in North-Indian tertiary care hospital. NZJ Lab Sci.

[9] Kumar S, Bandyoapdhyay M, Chatterjee M, Mukhopadhyay P, Poddar S, Banerjee P (2014). The first linezolid-resistant Enterococcus faecium in India: High level resistance in a patient with no previous antibiotic exposure. Avicenna J Med.

[10] Ruggero KA, Schroeder LK, Schreckenberger PC, Mankin AS, Quinn JP (2003). Nosocomial superinfections due to linezolid-resistant Enterococcus faecalis: evidence for a gene dosage effect on linezolid MICs. Diagn Microbiol Infect Dis.

[11] Egan SA, Shore AC, O'Connell B, Brennan GI, Coleman DC (2020). Linezolid resistance in Enterococcus faecium and Enterococcus faecalis from hospitalized patients in Ireland: high prevalence of the MDR genes optrA and poxtA in isolates with diverse genetic backgrounds. J Antimicrob Chemother.

[12] van de Veerdonk FL, Brüggemann RJ, Vos S (2021). COVID-19-associated Aspergillus tracheobronchitis: the interplay between viral tropism, host defence, and fungal invasion. Lancet Respir Med.

[13] Song G, Liang G, Liu W (2020). Fungal co-infections associated with global COVID-19 pandemic: a clinical and diagnostic perspective from China. Mycopathologia.

[14] Twilla JD, Finch CK, Usery JB, Gelfand MS, Hudson JQ, Broyles JE (2012). Vancomycin-resistant Enterococcus bacteremia: an evaluation of treatment with linezolid or daptomycin. J Hosp Med.

[15] Stefani S, Bongiorno D, Mongelli G, Campanile F (2010). Linezolid resistance in Staphylococci. Pharmaceuticals (Basel).

[16] Kamboj M, Cohen N, Gilhuley K, Babady NE, Seo SK, Sepkowitz KA (2011). Emergence of daptomycin-resistant VRE: experience of a single institution. Infect Control Hosp Epidemiol.

[17] Bender JK, Cattoir V, Hegstad K (2018). Update on prevalence and mechanisms of resistance to linezolid, tigecycline and daptomycin in enterococci in Europe: Towards a common nomenclature. Drug Resist Updat.

[18] Flamm RK, Mendes RE, Hogan PA, Streit JM, Ross JE, Jones RN (2016). Linezolid surveillance results for the United States (LEADER Surveillance Program 2014). Antimicrob Agents Chemother.

[19] Fowler T, Walker D, Davies SC (2014). The risk/benefit of predicting a post-antibiotic era: is the alarm working?. Ann N Y Acad Sci.

[20] Campion M, Scully G (2018). Antibiotic use in the intensive care unit: optimization and de-escalation. J Intensive Care Med.

[21] Kelly S, Collins J, Maguire M, Gowing C, Flanagan M, Donnelly M, Murphy PG (2008). An outbreak of colonization with linezolid-resistant Staphylococcus epidermidis in an intensive therapy unit. J Antimicrob Chemother.

[22] Lazaris A, Coleman DC, Kearns AM (2017). Novel multiresistance cfr plasmids in linezolid-resistant methicillin-resistant Staphylococcus epidermidis and vancomycin-resistant Enterococcus faecium (VRE) from a hospital outbreak: co-location of cfr and optrA in VRE. J Antimicrob Chemother.

[23] Mališová L, Jakubů V, Pomorská K, Musílek M, Žemličková H (2021). Spread of linezolid-resistant Enterococcus spp. in human clinical isolates in the Czech Republic. Antibiotics (Basel).

[24] Klare I, Fleige C, Geringer U (2015). Increased frequency of linezolid resistance among clinical Enterococcus faecium isolates from German hospital patients. J Glob Antimicrob Resist.

[25] Moure Z, Lara N, Marín M (2020). Interregional spread in Spain of linezolid-resistant Enterococcus spp. isolates carrying the optrA and poxtA genes. Int J Antimicrob Agents.

